# Immunological Responses and the Antioxidant Status in African Catfish (*Clarias gariepinus*) Following Replacement of Dietary Fish Meal with Plant Protein

**DOI:** 10.3390/ani11051223

**Published:** 2021-04-23

**Authors:** Rasha M. Reda, Mohammed A. F. Nasr, Tamer A. Ismail, Amira Moustafa

**Affiliations:** 1 Department of Fish Diseases and Management, Faculty of Veterinary Medicine, Zagazig University, Zagazig 44511, Egypt; 2 Department of Animal Wealth Development, Faculty of Veterinary Medicine, Zagazig University, El-Zeraa str. 114, Zagazig 44511, Egypt; nasr.maf@gmail.com; 3 Department of Clinical Laboratory Sciences, Turabah University College, Taif University, P.O. Box 11099, Taif 21944, Saudi Arabia; t.ismail@tu.edu.sa; 4 Department of Physiology, Faculty of Veterinary Medicine, Zagazig University, Zagazig 44519, Egypt; amiramostafa@zu.edu.eg

**Keywords:** *Clarias gariepinus*, *Aeromonas sobria*, fishmeal, soybean meal, sunflower meal

## Abstract

**Simple Summary:**

The price of feed ingredients is one of the most important and most costly constraints facing the aquaculture industry, and fishmeal is one of the most important and most expensive of these ingredients. As a result, recent research has focused on the use of alternative low-cost feed ingredients that are less reliant on fishmeal. Therefore, there has been considerable interest in the use of various types of plant protein (PP) meals in aquafeeds (soybean and sunflower meal). This study reveals that soybean and sunflower meal with methionine and lysine supplementation can be used on a larger scale in the aquafeed industry as substitutes of up to 50% of fishmeal.

**Abstract:**

African catfish (*Clarias gariepinus*) were subjected to a 30-day feeding trial to determine the appropriateness of using plant protein (PP) (soybean and sunflower meal) as a fishmeal (FM) replacement in the diet and its effects on immune status, antioxidant activity, pituitary adenylate cyclase-activating polypeptide (*PACAP*) gene expression, and disease resistance. A total of 150 *C. gariepinus* (51.01 ± 0.34 g) were randomly distributed among five groups in triplicate. Five experimental diets were formulated to replace 0 (control), 33.5, 50, 66.5, and 100% FM with soybean and sunflower meal to form the experimental diets (R0, R33.5, R50, R66.5, and R100, respectively). After 30 days, the diet containing PP for FM had no significant impact on total, and differential leukocyte counts determined at the end of the feeding period. The total globulin concentration showed significantly greater differences in the following order R0 > R33.5 > R50 > R66.5 > R100. The R0 group had the highest concentration of serum γ-globulin, while R100 had the lowest concentration. The antioxidant status complements 3 (C3), lysozyme activity (LYZ), and antiprotease activity were not significantly different between R0, R33.5, and R50 groups, while they were significantly lower in R100. The serum nitric oxide activity (NO) exhibited significantly greater differences in the following order R0 > R33.5 > R50 > R66.5 > R100. *PACAP* was significantly higher in the R33.5 group. The highest cumulative mortality caused by *Aeromonas sobria* was recorded in the R100 group (60%) and the lowest in the R0 group (30%). In conclusion, the results indicate that the immunological responses and antioxidant status of *C. gariepinus* were not affected when they consumed a diet with FM replaced by up to 50% with PP (SBM and SFM) with methionine and lysine supplementation, but total globulin, NO, and cumulative mortality were impaired with a diet containing a 100% FM replacement.

## 1. Introduction

The development of the fish industry and the expansion of aquaculture have become necessary internationally, particularly in developing countries, because of the nutritional, social, and economic value of fish. A settled source of fish is crucial for the nutrition and health of a wide segment of the globe [1]. Moreover, this sector provides employment and income in many developed and developing countries [2].

African catfish, *Clarias gariepinus* is a popular commercial fish species found in Africa, Europe, China, and Brazil, and is one of the most promising species for aquaculture production due to its distinct genetic and productive characteristics. *C. gariepinus* is mainly characterized by its high growth rate, which is estimated to be at least three times that of other fish species [3]. It can be raised in a high-intensity culture without daily water exchange because its airbreathing capacity allows it to withstand poor water quality and low dissolved oxygen [4]. Furthermore, *C. gariepinus* is distinguished by a wide range of tolerance to high salinity levels [5], the ability to withstand various stresses [6], high fecundity [7], and high palatability.

Achieving the highest profit percentage with the lowest cost percentage is critical to the growth of any industry. In the aquaculture industry, the prices of certain feed ingredients are among the major challenges affecting the industry’s success or failure. Among them, fishmeal (FM) is the main ingredient in aquafeed and the costliest [8,9]. FM, a conventional, highly palatable dietary protein source in aquafeed, is prepared from pelagic fish and contains balanced amino acids, essential n-3 fatty acids, vitamins, and minerals [2,10,11]. Over the last few years, researchers and fish feed manufacturers have increased their efforts to find the best alternative sources of FM, especially plant protein (PP) sources for various reasons, including a decline in pelagic and wild fish stocks leading to a shortage in their supply for FM production, and increased prices [12]. In addition, a greater proportion of pelagic species are increasingly being marketed as human food [2]. In addition, aquafeed consisting of more than 20% FM is hazardous to the ecosystem because the phosphorus content exceeds the requirements for fish and is released in urinary and fecal excretion, but PP contains less phosphorus than FM [13,14,15].

Therefore, there has been considerable interest in the use of various types of PP meals in aquafeed for the partial or total replacement of FM, such as soybean, cottonseed, pea seed, cassava leaf, sunflower, rapeseed meal, and rice protein meal [16,17,18,19,20,21]. Soybean meal (SBM) is the most widely used substitute protein source for fishmeal in fish and shrimp diets because it has many features and characteristics. SBM is readily available, inexpensive, rich in highly digestible protein with a balanced amino acid profile, easy to digest, and low in phosphorus compared to FM [22,23,24]. Although soybeans have some antinutritional factors, they can be reduced or eliminated through a variety of methods, including heat treatment during their manufacturing [25], via fermentation [26,27], the application of solvents [28], and alcohol extraction [29]. The cost of aquafeed can be further reduced by using other plant protein sources such as sunflower meal (SFM), which is less costly than FM and SBM [19]. SFM contains highly digestible protein with many amino acids (especially glutamic and aspartic acids) except lysine, and sulfur amino acids [30,31]. The essential amino acids that cannot be synthesized by the body, methionine and lysine, are obtained from the diet. Supplementing a fish diet with the recommended amounts of synthetic methionine and lysine will help minimize the oxidation of other amino acids and increase their utilization [32]. Dietary methionine and lysine are needed for protein, lipid, and energy metabolism, as well as carnitine biosynthesis [33]. Furthermore, the digestibility of feed ingredients is a vital factor that will indicate the nutritional balance of an aquatic animal’s diet [34]. Supplementation with amino acids, especially methionine and lysine, in diets high in plant protein ingredients has been shown to improve digestibility and, as a result, improve the growth and health of various fish species [35,36]. Previous research has also shown that any diet with unbalanced amino acid concentrations causes increased protein degradation [37].

*Aeromonadaceae* are an opportunistic Gram-negative bacterium that cause mass mortality and great economic losses in different fish species [38,39,40]. A balanced diet helps to protect fish from pathogens. Any imbalances resulting from the substitution of FM are therefore considered potent stressors that adversely affect fish welfare, immune status, and resistance to pathogens. Furthermore, several studies have shown that the duration of experimental diet administration had an impact on the immunological parameters, with immunological reactions often appearing in the first days of feeding (from 7 to 30 days) [41,42,43].

While FM replacement studies have mainly focused on growth, digestibility, and body composition, few have investigated the impact of this replacement on fish immunity, health, and disease resistance [19,44,45]. Accordingly, the current study was aimed at estimating the possibility of integrating PP (SBM and SFM) into practical diets *C. gariepinus* by replacing FM for 30 days and assessing its effects on immune status, antioxidant activity, pituitary adenylate cyclase-activating polypeptide (*PACAP*) gene expression, and disease resistance.

## 2. Materials and Methods

### 2.1. Ethical Statement

The experimental procedure was approved by the Ethics Committee of Institutional Animal Care and Use, Zagazig University, Egypt (ZU-IACUC/2/F/139/2020).

### 2.2. Fish

A total of 150 African catfish (51.01 ± 0.34 g) were procured from a private fish farm in Sharkia province, Egypt. The fish were transported in polyethylene bags filled with one-third dechlorinated water and two-thirds oxygen before sunrise to avoid heat and sunshine. When the fish arrived at the laboratory of the Fish Diseases and Management Department, Faculty of Veterinary Medicine, Zagazig University, Egypt, the water in the laboratory aquaria was slowly mixed over a period of time with the water the fish were transported in so as avoid a sudden change in water quality. To allow acclimation to the laboratory conditions, fish were randomly distributed in glass aquaria (80 × 40 × 30 cm, water capacity 60 L) for 14 days and fed twice daily with a basal diet (at 3% of biomass).

### 2.3. Experimental Diet and Design

Five isocaloric and isonitrogenous (32% crude protein) diets were formulated to replace 0 (control), 33.5, 50, 66.5, and 100% FM with plant protein sources (SBM and SFM) to form the experimental diets (R0, R33.5, R50, R66.5, and R100, respectively) [46,47] as shown in Table 1. The experimental diets were supplemented with methionine and lysine (as synthetic amino acids) and dicalcium phosphate to maintain the dietary requirements. The ingredients were mixed well and pelleted by passing them through a meat mincer (3 mm). This was then dried at room temperature and stored in the refrigerator (4 °C) during the feeding period. The proximate amino acid and chemical analyses were performed according to the Association of Official Agricultural Chemists [48] and Llames and Fontaine [49].

The fish were randomly distributed into 5 groups, and each group was in triplicate (30 fish/group with 10 fish/replicate). The first group (R0) was fed a control basal diet consisting of FM as the sole source of protein. The other 4 groups (R33.5, R50, R66.5, and R100) were fed experimental diets, in which FM was replaced by PP sources (SBM and SFM) for 30 days as mentioned in Table 1. The fish were fed manually at a rate of 3% from total fish biomass twice daily (08:00 and 15:00), and the quantity of food was adjusted every 2 weeks according to changes in fish body weight. Water parameters were kept at standard values throughout the experimental period (water temperature 27.5 ± 0.5 °C, pH 6.7 ± 0.2, ammonia 0.020 ± 0.001 mg/L, and nitrite 0.013 ± 0.003 mg/L). One-fourth of the volume of aquarium water was exchanged daily.

### 2.4. Sampling and Analytical Methods

#### 2.4.1. Blood Sample Collection

Blood samples were collected for serum separation after 15 and 30 days of experiment. At each point in time, 9 fish/group were collected and anesthetized with 100 mg/L of benzocaine solution (Al-Nasr pharmaceutical Chemicals Co., Oubour, Qalyubia, Egypt) [50]. Blood samples were collected from caudal vessels without anticoagulant for the separation of serum (centrifugation at 3000 rpm/15 min at 4 °C). The serum samples were held at −20 °C until analysis. For the analysis of total and differential leukocyte counts, the blood samples (9 fish/group) were collected after 30 days using EDTA-rinsed 1-mL syringes. After completing the blood sampling collection, the 9 fish were dissected to obtain samples for gene expression analysis, as explained below.

#### 2.4.2. Leukogram, Serum Total Protein, and Electrophoretic Fraction Analysis

Total and differential leukocyte counts were determined by using a Sysmex XT-2000iV Automated Hematology Analyzer (Sysmex Corporation, Hyogo, Japan) at the Animal Health Research Institute, Zagazig Branch, Egypt. Serum total protein (g/dL) [51] and albumin (g/dL) [52] were estimated, and total globulin was calculated by subtracting albumin from total protein. Serum protein was analyzed by sodium dodecyl sulfate-polyacrylamide gel electrophoresis (SDS-PAGE), according to Laemmli [53].

#### 2.4.3. Serum Oxidant/Antioxidant Status

Serum levels of total antioxidant capacity (TAC) (mM/L), superoxide dismutase (SOD) (U/mL), catalase activity (CAT) (U/L), and reduced glutathione (GPx) (mmol/L) were estimated using commercial ELISA test kits (Cusabio Biotech Co., Ltd., Wuhan, China) following the manufacturer’s instructions.

#### 2.4.4. Nonspecific Immune Analysis

Nonspecific immune parameters such as serum alternative complement pathway C3 activity were examined using a C3 kit (Zhejiang Elikan Biological Technology Co., Ltd., Wenzhou, Zhejiang, China). Reagent 1 was slowly blended with distilled water (2 mL), standard liquid, and serum. The absorbance was estimated at 340 nm after a 5-min incubation at 37 °C. The tubes were then filled with Reagent 2 and the absorbance was measured. A C3 standard curve was created using the same process. C3 levels were determined according to [54]. For serum nitric oxide (NO) (µmol/L), Griess reagent was applied to 100 mL of each serum sample in a microtiter plate and incubated at 27 °C for 10 min, then NO level was measured using a spectrophotometric method [55]. Using a turbidimetric assay, the activity of serum lysozyme (LYZ) (µg/mL) was determined by dissolving 0.75 mg/mL *Micrococcus lysodeikticus* (Sigma-Aldrich Chemie GmbH, Darmstadt, Germany ) in 0.1 M sodium phosphate buffer (pH = 5.9) and placing 175 mL of the suspension on a microtiter plate and incubating at 30 °C for 5 min. After that, 25 L of serum was applied to the microtiter plate. A lyophilized hen egg white lysozyme (Sigma) standard curve was used to measure the serum lysozyme levels (μg/mL) [56]. The antiprotease activity (mg/dL) was measured by incubating 10 mL of serum with 20 mL of trypsin solution (0.25% bovine pancreatic trypsin in 0.02% EDTA; Beyotime, Jiangsu, China) for 10 min at 25 °C. Then, 500 mL of 2 mM BAPNA (sodium-benzoyl-DL arginine-p-nitroanilide HCl; Himedia, Giza, Egypt) was added. TriseHCl (0.1 M, pH 8.2; Beyotime, China) was added to a final volume of 1 mL. The mixture was incubated at 22 °C for 25 min. The reaction was halted with 150 mL of 30% acetic acid, and the optical density (OD) was calculated at 415 nm against a blank using a BioRad microplate reader (Hercules, CA, USA). The inhibitory activity of the antiprotease was expressed as the percentage of trypsin inhibition, calculated as follows: ((optical density "OD" of trypsin blank_OD sample)/OD of trypsin blank) × 100. according to Bowden et al. [57].

#### 2.4.5. Expression of Pituitary Adenylate Cyclase-Activating Polypeptide Gene

The same 9 fish used for the collection of blood samples at the end of the experiment (30 days) were euthanized using a benzocaine solution overdose (250 mg/L) and used for gene expression analysis to collect spleen samples. The total RNA was extracted using the QIAamp RNeasy Mini Kit (Qiagen, Hilden, Germany, GmbH) according to the manufacturer’s protocol. Complementary DNA was synthesized following the manufacturer’s instructions (Quantitect^®^ Reverse Transcription kit, Qiagen, Germany). Quantitative real-time polymerase chain reaction (PCR) analysis was performed with the SYBR green PCR master mix (Step One Plus, Applied Biosystem, Waltham, MA, USA). Full-length elongation factor 1 alpha (EF-1α) (GenBank accession number AB075952.1) Available online: https://www.ncbi.nlm.nih.gov/nuccore/AB075952.1, (forward primer: 5′-CCTTCAACGCTCAGGTCATC-3′; reverse primer: 5′-TGTGGGCAGTGTGGCAATC-3′) was chosen as internal standard as suggested by Gröner et al. [58]. The target gene was *PACAP* (GenBank accession number = EF524513) Available online: https://www.ncbi.nlm.nih.gov/nuccore/EF524513, (Forward primer: 5′-CACTCGGACGGCATTTTCACGG-3′; Reverse primer: 5′–TTTGTTTCTAAACCTCTGTCTGTAC-3′) [59]. The amplification conditions were as follows: 40 cycles of 94 °C for 15 s, 65 °C for 30 s, and 72 °C for 30 s. The amplification efficiency of the used primer was determined by standard curve assay. Amplification efficiency was above 98% for each group.

### 2.5. Aeromonas sobria Challenge Test

Seven fish/replicate (21 fish/group) were challenged by intraperitoneal injection with 1.5 × 10^7^ cells/mL (adjusted with McFarland standard tubes) of *Aeromonas sobria* (*A. sobria*) previously isolated from moribund fish at the Department of Fish Diseases and Management, Faculty of Veterinary Medicine, Zagazig University (project no. 5589) and confirmed to be pathogenic. The fish were observed for 15 days, and their clinical signs, postmortem lesions, and daily mortality were recorded. For confirmation, the pathogenic bacterial strain was re-isolated from the liver, kidney, and intestine of dead fish.

### 2.6. Statistical Analysis

The data were first examined for homogeneity and normality. One-way ANOVA (SPSS version 16.0, IBM, Chicago, IL, USA) was performed to detect the difference among experimental groups within the same period. The group means were analyzed with Duncan’s multiple range tests and presented as the mean ± SE. Two-way ANOVA (Statistica software, version 8, StatSoft, Inc., 2008, Tulsa, OK, USA) was used to determine the interaction between the levels of FM substitution with PP and the feeding duration. For every test, the level of significance chosen was *p* ≤ 0.05.

## 3. Results

### 3.1. Leukogram, Serum Total Protein, and Electrophoretic Fraction

The data in Table 2 indicate that there were no significant differences in total and differential leukocyte counts of fish fed the experimental diets compared to the control group. Table 2 displays the concentrations of total serum proteins and their fractions. After 30 days of feeding, the highest total serum protein was recorded in the R0 and R50 groups, while there was no significant difference between R33.5, R66.5, and R100 in total serum protein. The total globulin concentration was significantly different in the order of R0 > R33.5 > R50 > R66.5 > R100. The concentrations of α1 and α2 globulin decreased significantly in the R100 and R66.5 groups compared to other groups, while the levels were not significantly different in R0, R33.5, and R50. The serum ß- globulin concentration was significantly higher in the serum of R0 and R33.5 groups, followed by the R50 group, while the lowest concentration was recorded in the R66.5 and R100 groups. The highest concentration of serum γ- globulin was recorded in the R0 group, and the lowest in the R100 group.

### 3.2. Serum Oxidant/Antioxidant Activity

**Figure 1:**
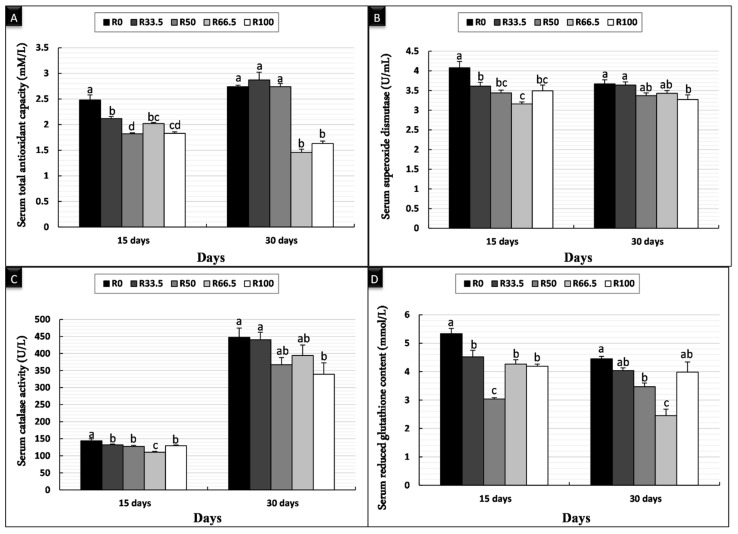
Serum antioxidant activity of *Clarias gariepinus* (mean ± SE) fed experimental diets in which fishmeal was replaced by plant protein (soybean and sunflower meal) after 15 and 30 days. (**A**) Bars indicate total antioxidant capacity (mM/L). (**B**) Bars indicate serum superoxide dismutase (U/mL). (**C**) Bars indicate serum catalase activity (U/L). (**D**) Bars indicate reduced serum glutathione content (mmol/L). Groups with different superscripts (a, b and c) were significantly different (*p* < 0.05, using one-way ANOVA to detect the difference among groups within the same period). R0 (control group) = fish fed normal base diet. R33.5, R50, R66.5, and R100 = fish fed diet, with fishmeal replaced by 33.5, 50, 66.5 and 100% plant protein (soybean and sunflower meal), respectively.

After 15 days of feeding, the serum TAC and GPx levels were significantly higher in the R0 group and significantly lower in the R50 group, while there was no significant difference in TAC in the groups that were fed substituted diets. SOD and CAT in serum were significantly higher in the R0 group and lowest in the R66.5 group. After 30 days of feeding, there was no significant difference in the TAC of the R0, R33.5, and R50 groups, which was significantly higher than that in R66.5 and R100 groups. After 30 days, the serum SOD and CAT levels were significantly higher in the R0 and R33.5 groups and significantly lower in the R100 group. The serum GPx level was significantly higher in the R0 group and significantly lower in the R66.5 group (Figure 1).

**Figure 2:**
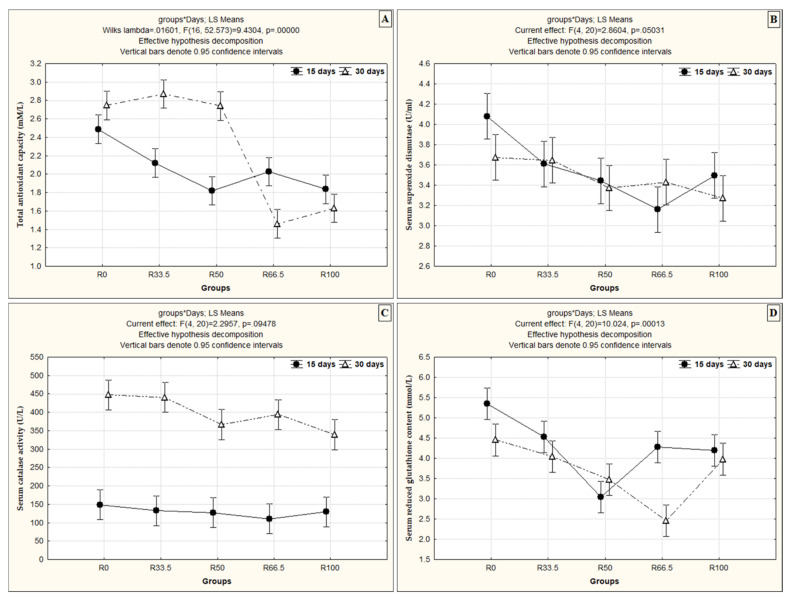
The interaction effect between the levels of fishmeal substitution with plant protein (soybean and sunflower meal) and feeding duration on (**A**) total antioxidant capacity (mM/L), (**B**) serum superoxide dismutase (U/mL), (**C**) serum catalase activity (U/L) and (**D**) serum reduced glutathione content (mmol/L). R0, R33.5, R50, R66.5 and R100: see legend of Figure 1.

The effects of the interaction between feeding duration and levels of FM substituted with PP were assessed, and the results reveal that, in all groups, the TAC level was dependent on time and level of FM substitution except in the R66.5 and R100 groups, where the TAC demonstrated better results after 15 days than 30 days of feeding (Figure 2A). This interaction between feeding duration and level of substituted PP had a different effect on the SOD level, which differed between groups, although it was not significantly different between R33.5 and R50 after 15 and 30 days. Over time, the SOD level decreased significantly in R0 and R100 and increased significantly in the R66.5 group (Figure 2B). There was a significant increase in serum CAT in all groups after 30 days compared to 15 days (Figure 2C). In contrast, the GPx level decreased significantly at the end of 30 days compared to 15 days of feeding in all groups except R50 (Figure 2D).

### 3.3. Nonspecific Immune Parameters

**Figure 3:**
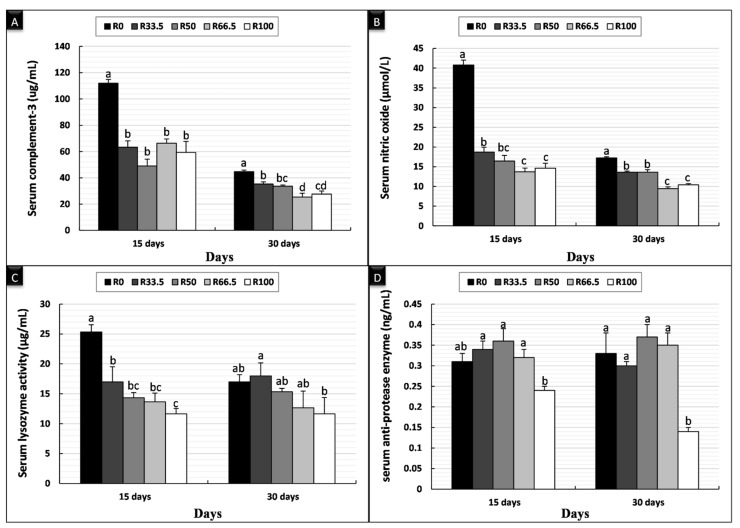
Nonspecific immune parameters of *Clarias gariepinus* (mean ± SE) fed experimental diets in which fishmeal was replaced by plant protein (soybean and sunflower meal) after 15 and 30 days. (**A**) Bars indicate serum complement-3 (μg/mL). (**B**) Bars indicate serum nitric oxide (μmol/L). (**C**) Bars indicate serum lysozyme activity (μg/mL). (**D**) Bars indicate serum anti-protease enzyme (ng/mL). Groups with different superscripts (a, b and c) were significantly different (*p* < 0.05, using one-way ANOVA to detect the difference among groups within the same period). R0, R33.5, R50, R66.5 and R100: see legend of Figure 1.

The nonspecific immune parameters after feeding *C. gariepinus* diets in which FM was substituted with PP are shown in Figure 3. After 15 days of feeding, serum C3 was significantly higher in the control group (R0) than the other experimental groups, and there was no difference between other groups. Serum NO and LYZ activity was significantly higher in R0 and significantly lower in R100, and there was no difference between other groups fed substituted diets, which were lower than R0. There was no significant difference in serum antiprotease activity among the groups. Similarly, after 30 days of feeding, serum C3 was significantly highest in R0, and there was no difference among other groups, which were lower than R0. Serum NO activity exhibited a significantly greater difference in the following order R0 > R33.5 > R50 > R66.5 > R100. Serum LYZ activity showed no significant differences between groups. Serum antiprotease activity decreased significantly in R100, and there was no significant difference between the other experimental groups.

**Figure 4:**
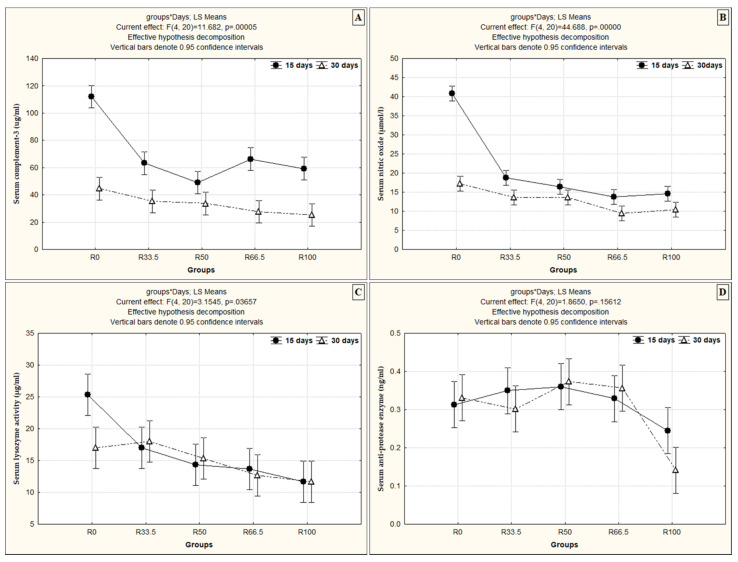
The interaction effect between the levels of fishmeal substitution with plant protein (soybean and sunflower meal) and feeding duration (*p* < 0.05, using two-way ANOVA) on (**A**) serum complement-3 (ug/mL), (**B**) serum nitric oxide (µmol/L), (**C**) serum lysozyme activity (µg/mL) and (**D**) serum anti-protease enzyme (ng/mL). R0, R33.5, R50, R66.5 and R100: see legend of Figure 1.

Serum C3 and NO activity in fish was significantly higher after 15 days than after 30 days of feeding on the substituted diet. Serum LYZ activity increased significantly with time and feeding on the substituted diet only in R33.5 and R50. Similarly, serum NO activity increased significantly only in R50 and R66.5 with time when fish were fed the substituted diets (Figure 4).

### 3.4. PACAP Gene Expression

**Figure 5:**
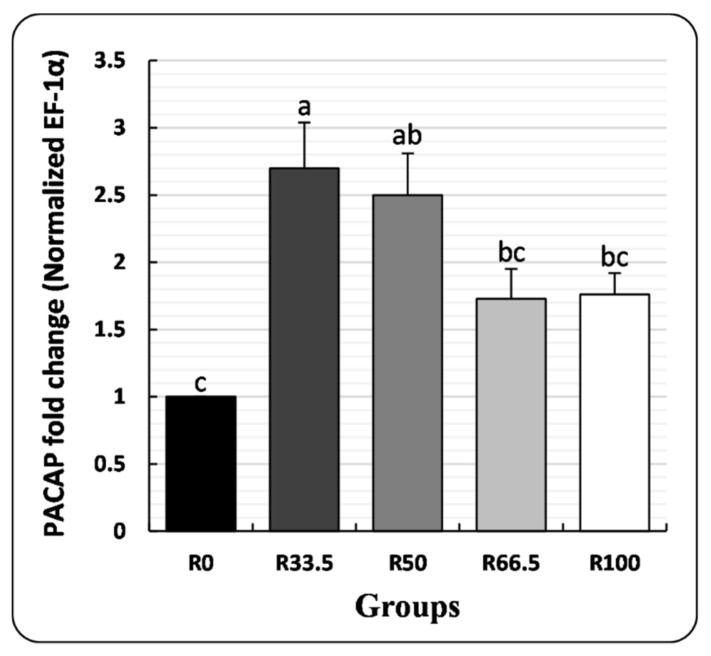
Pituitary adenylate cyclase-activating polypeptide (*PACAP*) gene expression in spleen of *Clarias gariepinus* (mean ± SE) fed experimental diets in which fishmeal was replaced by plant protein (soybean and sunflower meal) after 30 days. Groups with different superscripts (a, b and c) are significantly different (*p* < 0.05, using one-way ANOVA to detect the difference among groups within the same period). R0, R33.5, R50, R66.5 and R100: see legend of Figure 1.

*PACAP* cDNA was significantly higher in the R33.5 group and was not significantly different in other groups in comparison to R0 (Figure 5).

### 3.5. Resistance to Aeromonas sobria

**Figure 6:**
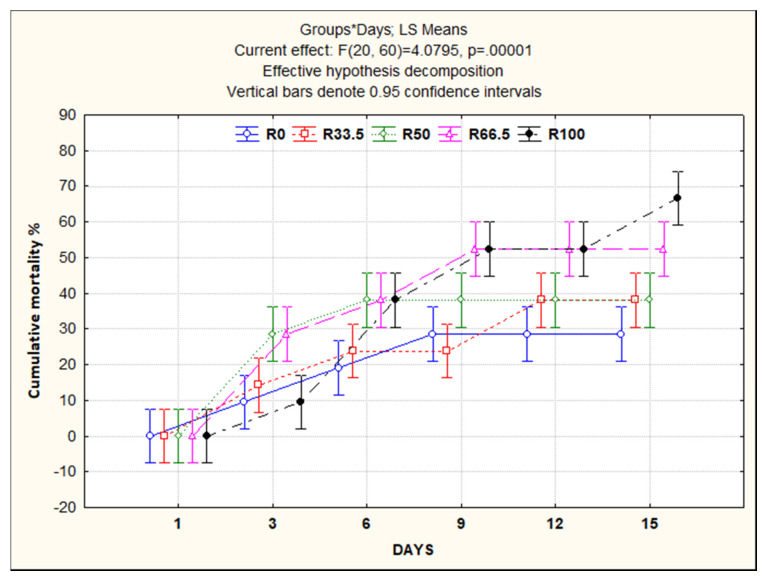
Cumulative mortality rate of *Clarias gariepinus* fed experimental diets in which fishmeal was replaced by plant protein (soybean and sunflower meal) after 30 days and challenged with pathogenic *Aeromonas sobria* for 14 days. R0, R33.5, R50, R66.5 and R100: see legend of Figure 1.

At the end of the feeding trial (30 days), the *C. gariepinus* were challenged with *A. sobria*. Different external clinical signs were recorded, such as sluggish movement and destruction of fin rays, especially dorsal and caudal fins, with hemorrhagic spots at the base. Internally, congestion and enlargement of internal organs, especially liver and kidney, were the main findings. The highest cumulative mortality was observed in the R100 group (60%), followed by R66.5 (50%), R33.5 and R50 (both 40%), and R0 with the lowest percentage (30%) (Figure 6).

## 4. Discussion

Aquafeeds incur high production costs; thus, the interest in using low-cost food processing by-products has increased. This new source of aquafeed production has reduced the need for expensive waste management systems and the reliance on expensive protein sources such as FM [60]. Not only does the wrong choice of protein source result in reduced weight gain and a longer time to reach marketable size, it also raises stress levels, leading to a declined physiological and immunological status of fish, with increased susceptibility to disease [24,61]. The current study, therefore, evaluated the effects of substituting FM with economic sources of PP on immune status, antioxidant activity, *PACAP* gene expression, and disease resistance.

Generally, the analysis of white blood cells and protein fractions serves as a diagnostic tool to measure the physiological status, nutritional condition, and health status of fish. Any changes in these parameters indicate an adverse condition contributing to stress on fish health [62,63,64]. The present study indicates that consuming a diet in which FM was substituted with PP did not have a significant effect on total and differential leukocyte counts. In an earlier study, a diet in which FM was replaced with PP sources (wheat gluten, corn gluten, and soybean meal) by 40, 70, and 100% did not significantly affect the blood parameters hematocrit, hemoglobin, white blood cell, heterophil, and lymphocyte counts of *Oncorhynchus mykiss* after 60 days of feeding [65]. Moreover, a diet in which SBM was replaced with SFM and sesame seed meal by 15, 30, and 45% had no significant effect on the leukocyte, eosinophil, monocyte, and lymphocyte counts of *C. gariepinus* [66]. Yue and Zhou [67] reported a substantial improvement in the white blood cell and hematocrit values of hybrid tilapia, *Oreochromis niloticus* × *O. aureus* when the fish consumed feed with cottonseed meal (CSM) replacing SBM from 0 to 45% but these values decreased dramatically when the amount of CSM was increased from 60 to 100%.

Antioxidant defense in fish involves enzymatic and non-enzymatic activities. The radical scavenging enzymes have different modes of action; some, such as SOD, act on superoxide (O^2–^), while others, such as CAT, act on hydrogen peroxide (H_2_O_2_), and glutathione peroxidase (GSH-PX) scavenges H_2_O_2_ and lipid hydroperoxides [68], which protects cells and tissues from oxidative damage. Antioxidant enzymes are important biomarkers of fish health and immune status [69]. Nutritional factors influence antioxidant defense in fish but, until now, there have been conflicting reports on the types and levels of dietary ingredients that exert such influence [70].

In the present study, no statistical difference in antioxidant activity was observed in the experimental groups, except for the dietary group receiving the lowest level of FM. Compared to the control group (R0), the antioxidant activity of the R66.5 and R100 groups diminished, and this corroborates with earlier research. *O. mykiss* spleen SOD and kidney CAT activities decreased significantly after feeding for 8 weeks on a diet with 20 and 30% of FM substituted with soy protein and meat bone meal [71]. Hepatic antioxidant activities (TAC, SOD, CAT, and GSH-Px) of *Pseudobagrus ussuriensis* decreased with increase in FM substituted with cotton meal, especially at 60% replacement after 8 weeks of feeding [72]. TAC in the serum of *Scophthalmus maximus* decreased significantly after feeding for 66 days on a diet with 45% of FM replaced by SBM [73]. CAT and TAC in the hepatopancreas of *Litopenaeus vannamei* decreased significantly in the group fed a low-FM diet (FM substitution of 15 to 25%) after 8 weeks of feeding [74]. Similarly, SOD activity decreased significantly in the hepatopancreas of *L. vannamei* fed a diet with 25% of FM substituted with SBM for 12 weeks [75].

The reduction in antioxidant index with a decrease in FM levels in the R66.5 and R100 groups indicates a disturbance in the antioxidant activity. This breaks the dynamics of free radical production and removal in fish, which could be linked to undegraded anti-nutritional factors (ANFs) in PP sources [72,73,74]. On the contrary, some studies have shown increased antioxidant activity in fish fed diet with FM substituted with a PP source [76,77,78,79,80], which they attributed to the presence of phenolic [76] and flavonoid [81] compounds in the plant constituents. These compounds could be stimulators increasing the antioxidant activity. A variety of factors such as feed ingredients and their levels, time, fish species, size, feeding behavior, and environmental factors, could reflected in the variations of antioxidant defense activity in fish [82].

The nonspecific immune system in fish is more important than the specific immune system in terms of tolerating diseases because the latter takes relatively more time to produce antibodies and induce specific cellular activations [83]. In our study, we focused on C3, NO, LYZ, and antiprotease activity. The complement system consists of 35 plasma proteins and plays a crucial role in innate and adaptive immunity by alerting and the host to the presence and the clearance of possible pathogens [84]. NO is normally derived from fish macrophages, which, due to its powerful killing effects and its function as a deactivator of some particular enzymes involved in macrophage cytotoxic reactions, is integral to fish antimicrobial immunity [85,86]. LYZ is a mucolytic enzyme of leucocytic origin that primarily inhibits the invasion of Gram-positive bacteria by breaking the cell wall linkages between N-acetylmuramic acid and N-acetylglucosamine [87,88]. Meanwhile, the blood protein antiproteases protect fish tissues from lysis by antagonizing the proteolytic activity of microorganisms [89]. No doubt, there is a close relationship between the dietary components, especially proteins, ingested by the organism and its immunity. Proteins consist of nitrogen, carbon, hydrogen, and oxygen and are present in the cell-foundation, enzymes, certain hormones, and the defense mechanism of any organism [90,91]. Besides the substantial role of protein in the defense mechanism of the immune system by producing natural and acquired immunity against pathogens, the immune systems relies on it for the development of active protein compounds and cell replication [91].

Some studies have investigated the impact of PP on fish immunity, and there are a few inconsistencies in their findings. The two main issues that lead to these discrepancies are the imbalance of important amino acids, especially methionine and lysine, whose levels are always below what is needed by the fish [92,93]. The second issue is the presence of ANFs in PP [93,94]. We believe that the different approaches to overcoming these two key issues caused the discrepancies in the findings on the impact of using PP on fish immunity, in addition to several other factors. Among the other factors, those that involve nutritional requirements include fish species, body size, feed ingredients other than PP, differences between fish species in handling and digesting various nutrients, and differences in environmental factors [42,80,95,96].

In the current study, after 15 days of feeding, the nonspecific immune parameters were significantly higher in R0 than in the other groups fed a diet with FM substituted by PP. By the end of the experiment (after 30 days), there was no significant difference between R0, R33.5, and R50 groups in C3, LYZ, and antiprotease activity, while R100 had significantly lower values for these parameters. Moreover, Jalili et al. [65] reported an insignificant effect on lysozyme activity and total antibodies in *O. mykiss* fed a diet with 40% FM substituted with PP sources (wheat gluten, corn gluten, and SBM) for 60 days. Alternative complement activity and total serum antibody were significantly lower in the groups fed diets with 70 and 100% FM substitution compared to the control [65]. Likewise, nonspecific immune responses were suppressed in *O. mykiss* when they consumed a diet containing more than 60% of FM substituted with soybean proteins for 53 days [97]. Serum LYZ of *S. maximus* decreased significantly in the group fed with 60% SBM for 66 days [73]. Moreover, the hemolymph phagocytic activity of *Macrobrachium nipponense* decreased when the proportion of FM decreased after 8 weeks of feeding [79]. Storebakken et al. [98] reported that the fish immune system was weakened when fed with high levels of PP, especially SBM, and attributed this to the decreased availability of vitamins and minerals, which are cofactors for metabolic enzymes, antioxidation, and the immune response.

*PACAP* is a regulatory neuropeptide that belongs to the glucagon superfamily [99]. A bidirectional relationship exists between the neuroendocrine system and its influence on immune functions. Neuropeptides are produced from the lymphoid tissue microenvironment, and corresponding immune cell neuropeptide receptors mediate neuroimmune interactions [100]. Some studies investigated the role of *PACAP* in the stimulation of innate and acquired immunity of fish [59,100,101]. However, to the best of our knowledge, this is the first study to investigate the effect of substituting FM with PP on *PACAP* gene expression. In the present study, the *PACAP* level was significantly higher in the R33.5 group, but there was no substantial difference between the other groups relative to R0. Mokrani et al. [80] indicated that dietary PP did not cause inflammation in blunt snout bream hepatopancreas and recorded a significant decrease in expression levels of interleukin 8 (IL-8), tumor necrosis factor alpha (TNF-a), and nuclear factor-kappa B (NF-_k_B) in FM-reduced groups. On the other hand, several intestinal transcriptomes of immune-related genes were upregulated in *Salmo salar* when they consumed a diet containing FM substituted with 20% SBM [102,103]. Moreover, Hedrera et al. [104] recorded an increase in the pro-inflammatory cytokine mRNA levels in *Danio rerio* larvae when they consumed SBM-containing diets at 50%, which may be related to SBM ANFs [103].

The bacterial challenge test is mostly used after the feeding trial as a final measure of fish health status [105]. *A. sobria* is a hemorrhagic septicemic Gram-negative bacterium that has been used as an indicator of fish immunonutrition in some studies [106,107]. In the present study, the highest and lowest percentage of cumulative mortality caused by *A. sobria* were recorded, respectively, for the R100 group at 60% and the R0 group at 30%. The results of cumulative mortality in this study could be confirmed by the immune parameter studies (Figure 3). Jalili et al. [65] recorded no major change in the mortality rate of *O. mykiss* consuming FM diet meal for 60 days, when 0, 40, 70, and 100% FM was substituted with PP (wheat gluten, corn gluten, and SBM). The mortality rates of *S. salar* infected with *Vibrio anguillarum* were not affected after 56 days of feeding on a diet with FM replaced with dehulled lupin meal [44]. Wedemeyer and Ross [108] reported that consuming a diet with FM substituted with PP (maize gluten or cottonseed meal) did not affect corynebacterial kidney disease infection in *O. mykiss*. On the other hand, Ding et al. [79] reported that cumulative mortality in *M. nipponense* increased significantly when they consumed a diet with decreased FM percentage and increased fermented SBM.

**Figure 7:**
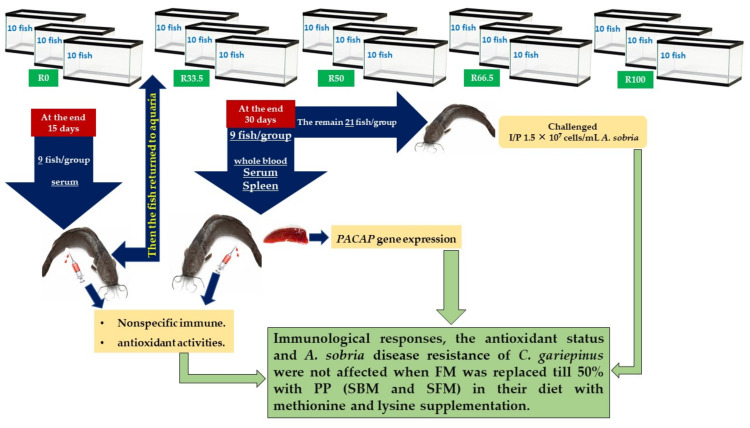
Summary of immunological responses and antioxidant status of *Clarias gariepinus* after feeding dietary fishmeal replaced by plant protein for 30 days (R0, control group = fish fed normal base diet. R33.5, R50, R66.5, and R100 = fish fed diet, with fishmeal replaced by 33.5, 50, 66.5 and 100% plant protein (soybean and sunflower meal), respectively).

From the aforementioned results, it can be said that up to 50% fishmeal in the diets for *C. gariepinus* could be replaced by plant protein (soybean and sunflower meal) along with supplementation of methionine and lysine without producing any significant differences in the immunological responses and antioxidant status (Figure 7). We used nonspecific immune system parameters in this study because they are considered the first line of protection for fish and are more critical for disease tolerance than a particular immune system that takes longer to activate [109]. The activity of the nonspecific immune response appeared in the resistance to *A. sobria*, which was considered pivotal in reducing cumulative mortality to 40% in R33.5 and R50. Furthermore, a recent study reported that replacing FM with soybean and sunflower meal with the addition of methionine and lysine in catfish resulted in comparable growth and body composition as well as greater economic performance when compared with the control group [17]. Protein digestibility was found to be better or equivalent to the reference diet with lysine and methionine + cystine supplementation, and up to 50% replacement of fishmeal protein with fermented linseed meal protein for rohu fingerlings [35].

## 5. Conclusions

Fishmeal is the costliest component of aquafeed. Reducing the amount of fishmeal in the diet without reducing the performance of fish is beneficial for fish production. The current study concludes that the replacement of fishmeal with a plant protein source (soybean and sunflower meal) by up to 50%, alongside methionine and lysine supplementation, showed a similar immune response with good disease resistance, as well as a decrease in the mortality rate. As such, this type of supplementation is recommended for use in the breeding and production of African catfish.

## Figures and Tables

**Table 1:** Ingredient composition and calculated nutrient content values of the experimental diets (% dry matter).

Ingredient	Experimental Diets (g/kg)				
	R0	R33.5	R50	R66.5	R100
Fishmeal 60%	150	100	75	50	0
Soybean meal 48%	411	453	475	509	500
Sunflower meal 36%	0	50	71	81	100
Wheat bran 14.5%	128	68	38	0	0
Ground yellow corn	286	303	311	323	303
Corn gluten 60%	0	0	0	0	50
Fish oil	20.0	20.7	22.4	24.5	25.0
Dicalcium phosphate	0	0	2	06.6	15.0
Vitamin mineral premix *	3	3	3	3	3
Dl-methionine	2.0	2.3	2.3	2.5	2.7
L-Lysine	0	0	0.3	0.4	1.3
Calculated composition (%Dry matter) **					
Crude protein	32.20	31.90	32.20	32.10	32.20
Crude fiber	3.75	3.95	3.98	4.05	4.12
Starch	21.10	21.00	20.80	21.00	20.90
Ether extract	5.35	5.11	5.13	5.14	5.12
Lysine	1.89	1.88	1.86	1.89	1.87
Methionine	0.80	0.80	0.79	0.78	0.79
Cysteine	0.42	0.41	0.43	0.43	0.42
Threonine	1.23	1.24	1.24	1.25	1.26
Arginine	2.26	2.29	2.30	2.30	2.28
Histidine	0.74	0.78	0.80	0.82	0.85
Isoleucine	1.33	1.40	1.43	1.45	1.49
Leucine	2.43	2.52	2.56	2.61	2.96
Phenylalanine	1.44	1.55	1.59	1.64	1.77
Tyrosine	1.09	1.10	1.11	1.13	1.21
Tryptophan	0.39	0.42	0.43	0.44	0.45
Valine	1.60	1.69	1.73	1.76	1.82
Calcium	0.99	0.77	0.69	0.68	0.63
Available P	0.53	0.39	0.35	0.35	0.35
DE (kcal/kg diet) ***	2660	2656	2658	2659	2662

R0 (control group) = fish fed normal base diet. R33.5, R50, R66.5, and R100 = fish fed diet with fishmeal replaced by 33.5, 50, 66.5 and 100% plant protein (soybean and sunflower meal), respectively. * Vitamin mineral premix kg ^−1^ diet: vit. A 8050 IU, vit. D3 2100 IU, vit. E 300 mg, vit. k3 14 mg, vit. C. 294 mg, vit. B1 19.6 mg, vit. B2 30.1 mg, vit. B6 14.7 mg, vit. B12 0.02 mg, Ca-D-biotin 0.2 mg, folic acid 0.4 mg, choline HCl 1.0 g, inositol 3000.0 mg, pantothenic acid 50.0 mg, nicotinic acid 100 mg, P-amino benzonic acid 50.0 mg. Mineral mix: each kg contained manganese 60 g, iron 80 g, copper 5 g, zinc 40 g, selenium 0.15 g and iodine 0.35 g.** All compositions calculated [46]. *** Digestible energy (DE) was calculated by applying the coefficient of 0.75 to convert gross energy to digestible energy according to Hepher et al. [47].

**Table 2:** Total and differential leukocyte counts and electrophoretic fractions of serum proteins of *Clarias gariepinus* (mean ± SE) fed experimental diets with fishmeal replaced by plant protein with methionine and lysine supplementation after 30 days.

Parameters	R0	R33.5	R50	R66.5	R100	*p*-Value
WBCs (10^3^/µL)	0.81 ± 0.034	0.78 ± 0.022	0.77 ± 0.056	0.75 ± 0.049	0.83 ± 0.060	0.749
Neutrophil (10^3^/µL)	0.003 ± 0.002 ^b^	0.013 ± 0.005 ^a,b^	0.030 ± 0.007 ^a,b^	0.030 ± 0.010 ^a,b^	0.040 ± 0.014 ^a,b^	0.063
Lymphocyte (10^3^/µL)	0.71 ± 0.07	0.71 ± 0.02	0.63 ± 0.05	0.66 ± 0.04	0.75 ± 0.04	0.466
Monocyte (10^3^/µL)	0.063 ± 0.021 ^a^	0.016 ± 0.005 ^c,d^	0.053 ± 0.010 ^a,b^	0.033 ± 0.01 ^a,b,c^	0.010 ± 0.006 ^d^	0.021
Eosinophil (10^3^/µL)	0.03 ± 0.015	0.02 ± 0.006	0.04 ± 0.010	0.02 ± 0.001	0.03 ± 0.015	0.718
Total protein (g/dL)	6.57 ± 0.09 ^a^	5.73 ± 0.06 ^b^	6.74 ± 0.10 ^a^	5.62 ± 0.06 ^b^	5.55 ± 0.03 ^b^	<0.001
Albumin (g/dL)	3.80 ± 0.05 ^c^	3.42 ± 0.09 ^d^	4.49 ± 0.03 ^b^	4.41 ± 0.05 ^b^	4.85 ± 0.05 ^a^	<0.001
Total globulin (g/dL)	2.77 ± 0.15 ^a^	2.31 ± 0.16 ^b^	2.25 ± 0.09 ^b^	1.21 ± 0.06 ^c^	0.70 ± 0.15 ^d^	<0.001
α1-globulin (g/dL)	0.49 ± 0.020 ^a^	0.48 ± 0.060 ^a^	0.50 ± 0.015 ^a^	0.25 ± 0.015 ^b^	0.16 ± 0.020 ^b^	<0.001
α2-globulin (g/dL)	0.70 ± 0.02 ^a^	0.70 ± 0.06 ^a^	0.73 ± 0.06 ^a^	0.33 ± 0.01 ^b^	0.15 ± 0.02 ^c^	<0.001
ß-globulin (g/dL)	0.74 ± 0.04 ^a^	0.65 ± 0.02 ^a^	0.52 ± 0.06 ^b^	0.31 ± 0.08 ^c^	0.20 ± 0.03 ^c^	<0.001
γ-globulin (g/dL)	0.82 ± 0.09 ^a^	0.46 ± 0.04 ^b^	0.49 ± 0.03 ^b^	0.3 1 ± 0.06 ^b,c^	0.18 ± 0.04 ^c^	<0.001

R0, R33.5, R50, R66.5 and R100: see legend of Table 1. WBCs: white blood cells. Values with different superscripts within the same row (^a^, ^b^, ^c^, and ^d^) were significantly different (*p* < 0.05, using one-way ANOVA).

## Data Availability

All data sets collected and analyzed during the current study are available from the corresponding author on fair request.
